# Risk-Stratified Screening for Perinatal Depression and Anxiety: Integrating Sexual Function, Self-Esteem, and Psychosocial Context

**DOI:** 10.3390/diagnostics16030412

**Published:** 2026-01-28

**Authors:** Roxana Ana Maria Dinescu, Alexandru Catalin Motofelea, Paul-Manuel Luminosu, Mihai Loichita, Nadica Motofelea, Ioan Sas

**Affiliations:** 1 Department of Doctoral Studies, “Victor Babeș” University of Medicine and Pharmacy Timișoara, Eftimie Murgu Square No. 2, 300041 Timișoara, Romania; roxana.dinescu@umft.ro (R.A.M.D.); paul.luminosu@umft.ro (P.-M.L.); nadica.motofelea@umft.ro (N.M.); 2 Department of Obstetrics and Gynecology, “Victor Babeș” University of Medicine and Pharmacy, 300041 Timisoara, Romania; sas.ioan@umft.ro; 3 Center for Molecular Research in Nephrology and Vascular Disease, Faculty of Medicine, “Victor Babeș” University of Medicine and Pharmacy, 300041 Timisoara, Romania; 4 Clinic of Anaeshtesia and Intensive Care, Emergency County Hospital Pius Brinzeu, 325100 Timisoara, Romania; 5 Department of Obstetrics and Gynecology, Timis County Emergency Clinical Hospital ‘Pius Brînzeu’, 300723 Timisoara, Romania; mihailoichita@gmail.com

**Keywords:** depression, anxiety, pregnancy, postpartum, social support, telemedicine

## Abstract

**Background**: Perinatal depression and anxiety are common but often under-detected. Current screening relies on depression-centered instruments and may miss relational drivers including sexual dysfunction, low self-esteem, and psychosocial adversity. **Objective**: To synthesize evidence on sexual function, self-esteem/body image, and psychosocial context as correlates of perinatal depression and anxiety, and propose a risk-stratified screening framework. **Methods**: We conducted a narrative evidence synthesis of studies from January 2010 to May 2025 (PubMed/MEDLINE, Scopus, Web of Science) examining associations between perinatal mood/anxiety outcomes and sexual function (Female Sexual Function Index), self-esteem/body image (Rosenberg Self-Esteem Scale), and psychosocial factors (perceived support, intimate partner violence). **Results**: Sexual dysfunction was highly prevalent and consistently associated with depressive and anxiety symptoms. Longitudinal evidence demonstrated bidirectional pathways: mood symptoms reduced sexual satisfaction, while sexual difficulties intensified relational strain and symptom persistence. Low self-esteem and negative body image mediated links between physiological changes and postpartum depression. Psychosocial adversity, particularly low partner support and intimate partner violence, identified high-risk subgroups with greater severity and slower recovery. Single-instrument approaches (Edinburgh Postnatal Depression Scale alone) may miss pregnancy-specific anxiety and postpartum relational drivers. **Conclusions**: A staged, risk-stratified model is recommended: assess pregnancy-specific anxiety alongside depression screening in the second/third trimesters; postpartum, selectively add sexual function and self-esteem assessment for women with elevated symptoms or psychosocial risk. Integration within defined referral pathways may improve detection and enable targeted perinatal mental health care.

## 1. Introduction

Significant physical, hormonal, and psychosocial changes occur throughout pregnancy and the transition to motherhood. These changes may substantially affect maternal well-being, fetal development, and overall quality of life, even in otherwise uncomplicated pregnancies [1,2,3]. Consequently, women are particularly vulnerable to mental health disturbances during the perinatal period, with depression and anxiety representing the most frequently reported conditions during pregnancy and the postpartum stage [4]. While stress is a common aspect of daily life, excessive or persistent stress during pregnancy may impair emotional regulation, sleep quality, appetite, and functional capacity, thereby increasing vulnerability to adverse mental health outcomes [5,6]. Furthermore, untreated mental health issues during pregnancy have been associated with adverse results for the fetus and the mother [4].

Epidemiological data indicate that perinatal mental health disorders constitute a major global public health concern. Depressive and anxiety symptoms may emerge during pregnancy as a result of hormonal fluctuations, sleep disturbance, psychosocial stressors, and pre-existing vulnerability [7,8]. Data from Biresaw et al. shows that approximately 13.7% of pregnant women report elevated stress levels during pregnancy [9]. Moreover, postpartum depression (PPD) remains highly prevalent, with a recent meta-analysis reporting a global pooled prevalence of 14.0%, ranging from 5.0% to 26.3% across countries [10,11]. Higher prevalence rates have been observed in low- and middle-income settings, underscoring the influence of contextual and socioeconomic factors on maternal mental health [11].

Beyond established psychosocial determinants, growing evidence indicates that sexual functioning is closely linked to psychological well-being and relational adjustment during pregnancy and the postpartum period. Intimacy and satisfactory sexual functioning have been associated with lower levels of anxiety and depressive symptoms, whereas sexual dysfunction during pregnancy has been correlated with increased emotional distress [12,13]. In parallel, antenatal depression has been associated with prior depressive episodes, low perceived social support, relationship factors, unintended pregnancy, and exposure to violence, emphasizing the relevance of relational and psychosocial contexts in shaping perinatal mental health risk [14].

Given the established impact of maternal mental health on pregnancy outcomes and child development, early identification of psychological vulnerability during pregnancy is essential. Evidence suggests that perceived stress and related psychological symptoms should be systematically assessed, particularly during the first and third trimesters, to support timely recognition and management of perinatal depression and anxiety [8,9]. However, how relational domains such as sexual function and self-esteem might contribute to early risk identification and postpartum symptom persistence remains less clearly integrated into routine perinatal mental health assessment and screening strategies.

The aim of this narrative review is to synthesize current evidence on relational and psychological factors sexual function, self-esteem, body image, partner support, and intimate partner violence as predictors of perinatal depression and pregnancy-specific anxiety, and to propose a risk-stratified screening framework. This review delivers: (i) evidence synthesis across antenatal and postpartum time windows; (ii) assessment of differential risk factors emphasizing pregnancy-specific anxiety during gestation; and (iii) a staged screening algorithm integrating depression screening (EPDS), pregnancy-specific anxiety (PRAQ-R2), and selective postpartum evaluation of sexual function (FSFI), self-esteem (RSES), and partner support (MSPSS) for high-risk women.

## 2. Methodology

This manuscript presents a narrative evidence synthesis using a structured and transparent approach to summarize contemporary literature on relational and psychosocial determinants of perinatal depression and anxiety and their implications for risk-stratified screening. Given the substantial heterogeneity across study designs, populations, timing windows within the perinatal period, and measurement instruments, findings were synthesized narratively rather than through meta-analysis.

A comprehensive literature search was conducted in PubMed/MEDLINE, Scopus, and Web of Science to identify articles published between January 2010 and May 2025. Search strategies combined terms related to the perinatal period, mental health outcomes (including depression, anxiety, postpartum depression, and psychological distress), and relational or psychosocial exposures (including sexual function, sexual dysfunction, intimacy, self-esteem, body image, social support, and intimate partner violence). To capture evidence relevant to screening and clinical implementation, additional terms relating to screening, risk stratification, and commonly used assessment instruments were included Edinburgh Postnatal Depression Scale (EPDS), Patient Health Questionnaire-9 (PHQ-9), Generalized Anxiety Disorder-7 (GAD-7), Pregnancy-Related Anxiety Questionnaire-Revised 2 (PRAQ/PRAQ-R2), Female Sexual Function Index (FSFI), Rosenberg Self-Esteem Scale (RSES), Multidimensional Scale of Perceived Social Support (MSPSS), and Mini-International Neuropsychiatric Interview (MINI). Reference lists of eligible studies and key review articles were manually screened to identify additional relevant publications.

A sensitive search strategy was employed, with relevance determined during title/abstract and full-text screening. Keyword searching combined terms related to the perinatal period, mental health outcomes, and relational or psychosocial factors using the following structure: (pregnancy OR prenatal OR antenatal OR perinatal OR postpartum OR postnatal) AND (depression OR anxiety OR “postpartum depression”) AND (“sexual function” OR “sexual dysfunction” OR intimacy OR “self-esteem” OR “body image” OR “social support” OR “intimate partner violence”) AND (women OR maternal). Searches were conducted in PubMed/MEDLINE, Scopus, and Web of Science, with publication dates restricted to 1 January 2010 through 31 May 2025.

Only English-language publications were included. We acknowledge that this restriction may have excluded relevant studies published in other languages, particularly from non-English-speaking regions with substantial perinatal mental health research. However, given the pragmatic focus on screening tools predominantly validated in English-speaking settings and resource constraints related to manual translation, this limitation was considered acceptable for the current synthesis. No geographic restrictions were applied.

Database-specific field restrictions were used, including MeSH terms and Title/Abstract fields in PubMed, Title-Abstract-Keywords in Scopus, and Topic fields in Web of Science. Boolean operators and wildcard truncation (pregnant to capture pregnant, pregnancy, and pregnancies) were applied as appropriate for each database.

Records retrieved from the database searches were exported and deduplicated using automated reference-management software with manual verification. Titles and abstracts were independently screened by two reviewers (R.A.M.D. and A.C.M.) according to predefined inclusion criteria, with disagreements resolved through discussion and consultation with a third reviewer (I.S.) when necessary. Full-text articles deemed potentially eligible were independently assessed by the same reviewers. Studies were excluded at this stage if they did not address the perinatal period, did not examine depression or anxiety outcomes, did not assess at least one predefined relational or psychosocial exposure, lacked original outcome data (such as protocols or conference abstracts), or were published in languages other than English.

Data extraction was performed using a structured evidence matrix. One reviewer (R.A.M.D.) extracted data, which were subsequently verified by a second reviewer (A.C.M.). Extracted information included study characteristics (author, year, country, setting, design, and population), perinatal timing window, mental health outcomes and assessment instruments, relational or psychosocial exposures and corresponding measures, key effect estimates, and adjustment for potential confounders. Following full-text review, 127 studies met all inclusion criteria and were included in the final synthesis. The final sample comprised 89 observational studies (cross-sectional and cohort designs), 18 longitudinal studies, 14 systematic reviews and meta-analyses, and 6 implementation studies.

Studies were eligible if they were peer-reviewed original research, systematic reviews, or meta-analyses; involved pregnant individuals or women up to 12 months postpartum; examined depression and/or anxiety outcomes or screening performance; and addressed at least one domain relevant to the proposed framework, including pregnancy-specific anxiety, sexual function or intimacy, self-esteem or body image, social support or partner context, or intimate partner violence. Use of validated instruments or structured diagnostic assessments was required where applicable. Editorials, commentaries, protocols without outcome data, non-peer-reviewed literature, animal studies, and studies not focused on maternal perinatal mental health were excluded.

Extracted evidence was organized into three thematic domains: environmental and psychosocial context (including access barriers, rurality, social support, and intimate partner violence); relational and psychological markers (sexual function, intimacy, self-esteem, and body image); and screening and implementation characteristics (including screening timing, instrument combinations, diagnostic performance, and linkage-to-care metrics). Because this was a narrative synthesis, a formal risk-of-bias tool was not applied across all study designs. Instead, interpretive weight was guided by study design, with greater emphasis placed on prospective and longitudinal studies, as well as sample size, measurement validity, clarity of perinatal timing, and adjustment for confounding factors. Findings were synthesized narratively, emphasizing consistent patterns and clinically relevant implications for staged, risk-stratified perinatal mental health screening.

## 3. Results and Discussion

### 3.1. Environmental and Psychosocial Context as Determinants of Risk and Under-Detection

Perinatal depression and anxiety are common and clinically consequential, with associations with adverse obstetric outcomes and impaired mother–infant bonding [15,16,17].

While biological factors contribute, evidence indicates that contextual determinants particularly geographic access to care and psychosocial resources shape both true symptom burden and the likelihood that symptoms are recognized and addressed during routine maternal care [18,19]. Accordingly, environmental and psychosocial exposures are best conceptualized as drivers of risk accumulation and screening failure, rather than as isolated correlates.

#### 3.1.1. Rurality, Access Barriers, and Delayed Care

**Figure 1:**
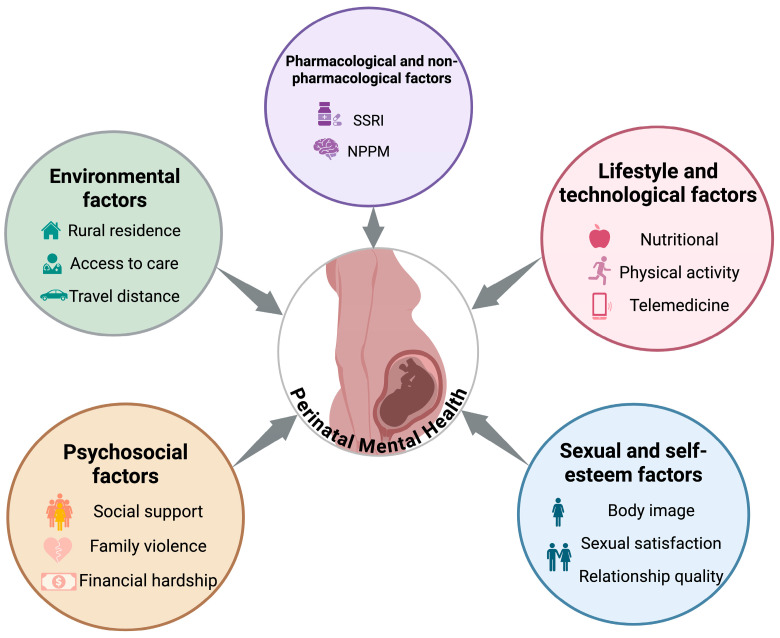
Conceptual framework shaping perinatal depression and anxiety.

Women living in rural areas often face challenges during and after pregnancy that can increase the risk of anxiety and depression symptoms (Figure 1) [20].

In the United States, gaps in maternal healthcare remain a serious concern. Recent data show that more than one-third of U.S. counties lack basic maternity services, and nearly 60% of these underserved areas are located in rural regions [21]. These geographic inequities have intensified over time due to closure of maternity units and a decreasing proportion of family physicians providing obstetric services, contributing to fewer points of contact for antenatal monitoring and mental health screening.

Similar access barriers are reported globally. In many low- and middle-income countries, long travel distances, limited transportation, poor infrastructure, and financial constraints reduce antenatal visit frequency and amplify psychological strain [22,23].

European data likewise suggest lower availability of midwifery and perinatal mental health services in rural compared with urban areas [24]. Although rural communities may benefit from strong informal support networks, these rarely compensate fully for structural deficits in service access.

Importantly, reduced access translates into delayed or absent care, which has direct implications for detection of perinatal mental health conditions. Evidence from a cross-sectional survey of pregnant and postpartum women indicates higher prevalence of anxiety and depression among those reporting delayed maternity care compared with those receiving timely services [25]. These findings support a clinical pathway in which delayed access increases uncertainty about pregnancy and fetal well-being, elevates stress, and reduces opportunities for early identification and intervention.

Where local maternity services are absent, women often travel long distances for obstetric care, creating additional financial burden—particularly for socioeconomically disadvantaged groups—due to transport and accommodation costs [26,27,28]. Beyond economic strain, travel requirements may increase physiologic risk and psychological distress; travel to emergency obstetric care has been associated with higher maternal mortality in some settings [26,29]. Quantitative work using the Rural Pregnancy Experience Scale (RPES) shows that each additional 10 min of travel time is associated with a 0.60-point increase in pregnancy-related stress, reinforcing distance as a measurable driver of anxiety risk [30]. Consistent with this, women living in rural areas without local maternity care facilities may face substantially longer journeys to reach obstetric hospitals compared with those in full-access regions, contributing to increased stress and poorer maternal outcomes [21]. Relocation or prolonged absence from family support networks may further increase isolation and exacerbate anxiety and depressive symptoms during a vulnerable period [31].

Stigma and low mental health literacy may further compound rural disparities. Compared with urban settings, rural communities may exhibit stronger norms of self-reliance and greater stigma surrounding mental illness, reducing disclosure and help-seeking [32,33,34]. Limited referral pathways and fewer mental health resources can also restrict access even among women motivated to seek care [35]. Supporting an overall rural disadvantage, a UK cross-sectional study using EPDS, Whooley questions, and GAD-2 reported higher odds of depression and anxiety among rural compared with urban perinatal women, with higher prevalence in rural areas (40% vs. 28.5%) [36]. Collectively, these findings indicate that rurality is linked not only to higher risk but also to a higher likelihood of under-recognition and undertreatment, which is directly relevant to screening design.

#### 3.1.2. Social Support, Partner Factors, and Psychosocial Adversity

Psychosocial support is consistently associated with lower perinatal anxiety and depressive symptoms [16,37,38,39,40]. Social support from family and peers may reduce isolation, provide reassurance, and buffer stressors related to pregnancy and early parenting [39,41,42]. Conversely, low perceived support is associated with substantially higher psychological distress, including a reported threefold increase in vulnerability among women with low support compared with those reporting high support [42,43]. Studies using MSPSS show negative associations between perceived support and symptoms of anxiety, depression, and stress, with lower family and friend support linked to higher depressive and stress symptoms [44]. Notably, perceived availability of support may be more relevant than objective support exposure, suggesting that screening should prioritize women’s appraisal of support resources [43]. This is further supported by evidence associating higher pregnancy-related anxiety scores with lower MSPSS scores, indicating that reduced perceived support aligns with elevated anxiety risk [45].

Partner support represents a particularly salient protective factor. Involvement in antenatal visits, shared household responsibilities, emotional reassurance, and practical support can strengthen maternal coping and reduce loneliness, functioning as a buffer against depression and anxiety during pregnancy and postpartum [16,46,47,48,49,50]. In contrast, partner emotional neglect and low involvement are associated with increased risk of depressive symptoms [40,51].

At the severe end of psychosocial adversity, intimate partner violence (IPV)—physical, sexual, or psychological—has repeatedly been linked to poor perinatal mental health outcomes, including depression, anxiety, and PTSD [52,53,54]. A systematic review reported that IPV may increase risk of antenatal depression up to threefold and postpartum depression up to sevenfold, depending on type and severity [55]. Prospective cohort evidence from Tanzania found that women exposed to violence had more than three times the odds of postpartum depression [56], and longitudinal data from Turkey similarly identified violence as a key predictor of postpartum depression [57]. Psychological abuse alone has also been associated with markedly elevated postpartum depression risk (6.5-fold), underscoring that non-physical violence carries substantial mental health consequences [58]. Given both the magnitude of association and actionability through safeguarding and referral pathways, IPV represents a high-yield target for routine perinatal screening.

Additional psychosocial stressors particularly unintended pregnancy and financial hardship further increase vulnerability. Unplanned pregnancy has been associated with higher risk of depression during pregnancy and persistence of depressive symptoms into the postpartum period, including evidence of approximately 2.5-fold increased risk up to 11 months postpartum in a Brazilian prospective cohort [59,60,61]. Economic insecurity and material hardship, including food insecurity, have also been associated with higher prenatal anxiety, stress, and depression [62,63]. These exposures may act synergistically by increasing interpersonal conflict and reducing capacity to access supportive resources, thereby sustaining psychological distress into the postpartum period [64].

### 3.2. Sexual Function & Self-Esteem

**Figure 2:**
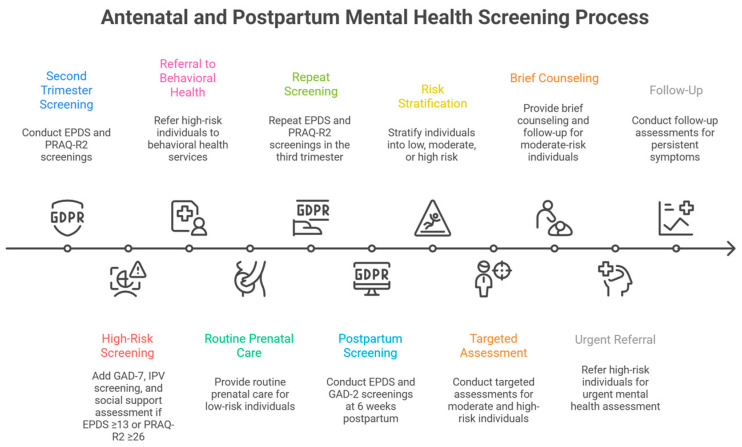
Proposed risk-stratified staged screening framework integrating pregnancy-specific anxiety, depression, psychosocial risk factors, and selective postpartum assessment of sexual function, self-esteem, and partner support for high-risk women. See text and Table 1 and Table 2 for supporting evidence. Abbreviations: EPDS, Edinburgh Postnatal Depression Scale; FSFI, Female Sexual Function Index; GAD, Generalized Anxiety Disorder scale; IPV, intimate partner violence; MSPSS, Multidimensional Scale of Perceived Social Support; PRAQ-R2, Pregnancy-Related Anxiety Questionnaire-Revised 2; RSES, Rosenberg Self-Esteem Scale.

Marital and relationship dissatisfaction frequently emerge during pregnancy and may persist for more than one year postpartum, with important implications for maternal psychological well-being [65]. One prominent manifestation of relational strain during the perinatal period is impaired sexual functioning, which represents a central component of women’s emotional health and overall quality of life [66]. Sexual function during pregnancy and after childbirth is shaped by both relational context and biological changes. Hormonal fluctuations involving estrogen, progesterone, and prolactin may alter libido, mood regulation, and orgasmic response, while physical discomfort, fatigue, sleep disruption, weight gain, and body image changes further contribute to reduced sexual desire and activity [67,68,69]. Breastfeeding-related dyspareunia and vaginal dryness may additionally exacerbate sexual difficulties in the postpartum period [70,71] (Figure 2).

Sexual intimacy is known to promote emotional closeness and stress reduction through neurobiological mechanisms, including endorphin release, which may buffer psychological distress [13]. Consequently, reduced or absent sexual intimacy during pregnancy and postpartum may weaken an important protective factor against anxiety and depressive symptoms. Empirical evidence indicates that sexual dysfunction is highly prevalent during pregnancy. A cross-sectional study reported that 92% of women experienced some degree of sexual dysfunction in the third trimester, accompanied by a progressive decline in Female Sexual Function Index (FSFI) scores as pregnancy advanced [72]. These findings are consistent with multiple studies documenting significantly lower sexual desire, arousal, and satisfaction among pregnant women compared with non-pregnant controls [68,71,73,74].

Beyond prevalence, growing evidence highlights a close and potentially bidirectional relationship between sexual dysfunction and perinatal mental health. Longitudinal data from Wallwiener et al. demonstrated that lower FSFI scores in late pregnancy were significantly associated with higher EPDS scores up to four months postpartum, indicating that impaired sexual functioning tracked depressive symptom severity over time [71]. Other studies similarly report that women experiencing depression during pregnancy or postpartum are more likely to develop persistent sexual dysfunction, with sexual desire continuing to decline for up to six months after delivery [75]. Neurobiological research suggests that depressive states may reduce emotional intimacy and alter neural circuits involved in sexual arousal, further reinforcing this reciprocal relationship [76]. Collectively, these findings suggest that sexual dysfunction may function both as a consequence of perinatal depression and anxiety and as a contributing factor to symptom persistence through relational strain and reduced perceived support.

Self-esteem represents a closely related psychological domain that may further amplify vulnerability. An unsatisfying sexual life and negative changes in body image can undermine self-esteem, which in turn has been associated with higher levels of anxiety and depressive symptoms during the perinatal period [77]. Low self-esteem is frequently linked to distorted body image and negative self-appraisal, particularly in the context of pregnancy-related physical changes [68,78]. Evidence using the Rosenberg Self-Esteem Scale (RSES) indicates that lower self-esteem scores are correlated with greater body dissatisfaction and increased vulnerability to anxiety symptoms during pregnancy [78]. Postpartum studies further demonstrate that women with low self-esteem are substantially more likely to experience depressive symptoms, with some reports indicating markedly elevated risk compared with women reporting high self-esteem [79].

Taken together, these findings position sexual function and self-esteem as salient relational–psychological markers that intersect with perinatal depression and anxiety. Rather than representing secondary consequences of mood disturbance alone, impairments in sexual functioning and self-esteem may contribute to ongoing relational stress and reduced emotional support, thereby sustaining psychological distress into the postpartum period. These domains may therefore hold clinical relevance for identifying women at risk of persistent symptoms who may not be fully captured by depression-focused screening instruments alone.

### 3.3. Lifestyle and Digital Health Tools as Adjuncts to Perinatal Mental Health Care

Artificial intelligence-based prediction models have emerged as a potential tool for early risk stratification in perinatal mental health.

The evidence base for AI-based postpartum depression prediction has expanded rapidly, with comprehensive scoping reviews documenting growth from 14 studies in 2021 [80] to 65 studies by early 2025 [81]. Across this literature, supervised machine learning algorithms—particularly random forest, support vector machines, and logistic regression—demonstrate consistently high predictive accuracy, with area under the receiver operating characteristic curve (AUROC) values frequently exceeding 0.9 in controlled research settings [82,83]. Key predictive features identified across studies include maternal age, pregnancy stress and adverse emotions, mental health history, maternal education, marital relationship quality, and sleep status [82]. Counterintuitively, one large-scale analysis of the NIH nuMoM2b dataset (n = 8454) found that pre-pregnancy mental health conditions were not the most predictive factor, with body mass index, prior depression, age, and income demonstrating greater predictive utility [83]. However, translation to real-world clinical settings reveals substantial challenges. A tertiary care implementation study (n = 12,284) achieved a more modest AUC of 0.73, highlighting the performance gap between research and practice environments [84]. Critically, this study found that adding socioeconomic census-tract data did not improve prediction, underscoring the importance of individual-level data, and identified significantly lower model performance among Hispanic patients (AUC 0.71 vs. 0.74 in White patients), raising concerns about algorithmic equity [84]. Systematic limitations across the field include geographic bias toward high-income countries (27.7% US-only studies), minimal external validation, inconsistent reporting of performance metrics, limited deep learning adoption, reliance on unimodal structured data, and insufficient attention to algorithm interpretability and cross-cultural applicability [81,82]. These findings underscore that while AI-based prediction models demonstrate technical feasibility, successful clinical implementation will require robust external validation, attention to health equity, transparent algorithmic processes, and integration within existing clinical workflows rather than standalone deployment.

Beyond prediction algorithms, mobile health applications and telemedicine platforms have been explored as intervention and engagement tools to support maternal mental well-being during pregnancy and the postpartum period.

Mobile health (mHealth) applications, mindfulness-based programs, and telemedicine platforms have demonstrated potential to reduce anxiety and depressive symptoms and to improve perceived quality of life among pregnant women [85,86,87] offering accessible alternatives when in-person care is limited.

Evidence suggests that app-delivered mindfulness and digital psychoeducation may be particularly useful in enhancing emotional regulation and reducing distress, especially when access to in-person care is limited [85,86]. Telemedicine interventions implemented during the COVID-19 pandemic further highlighted the capacity of remote care models to alleviate pregnancy-related anxiety and health-related fears [87,88].

Importantly, user acceptability appears highest among women with higher educational levels or prior mental health engagement, indicating potential selection effects [89,90]. Despite their promise, digital tools are best viewed as complementary rather than standalone solutions. Concerns related to data privacy, usability, clinician workload, and unequal access remain significant barriers to widespread implementation [89,90,91].

Critically, both AI prediction models and digital therapeutic interventions exhibit a fundamental limitation: they do not directly assess or address the relational and psychological domains identified earlier as central risk factors. Current AI models predominantly incorporate sociodemographic, clinical, and obstetric features [81,82,84], with minimal integration of sexual function, self-esteem, body image, or dyadic relationship quality factors demonstrated in this review to predict symptom persistence and postpartum adjustment [71,92,93,94,95,96,97,98]. Similarly, app-based mindfulness and telemedicine platforms do not directly address partner support, sexual functioning, or self-esteem [89,90,91], which are increasingly recognized as central to perinatal mental health risk and symptom persistence.

### 3.4. Interaction Among Determinants of Perinatal Mental Health

Perinatal mental health is shaped by the dynamic interaction of multiple determinants rather than by isolated risk factors. Structural barriers such as limited access to healthcare, geographic distance from services, and financial stress may interact with psychosocial vulnerabilities, including low social support and relationship difficulties, resulting in cumulative risk of anxiety and depressive symptoms during pregnancy and the postpartum period [99]. Periods of heightened external stress, such as the COVID-19 pandemic, have illustrated how rapidly these interacting pressures can amplify perinatal mental health burden [100].

Relational and psychological domains play a central role within this network of influences. Changes in body image, sexual functioning, and self-esteem during and after pregnancy may reinforce one another and contribute to emotional distress, particularly when combined with reduced partner support or social isolation [99]. Given that anxiety symptoms affect up to one in five pregnant women [101], failure to account for these interacting domains may result in under-recognition of clinically meaningful distress.

Digital health tools and telemedicine have emerged as potential mechanisms to mitigate some access barriers and maintain continuity of care. However, benefits remain unevenly distributed, with women in urban or higher-income settings more likely to engage with and benefit from these technologies [102,103]. Consistent with integrated care models, evidence suggests that perinatal mental health outcomes are optimized when emotional, relational, and physical health needs are addressed together rather than in isolation [104]. While telehealth may improve engagement and reduce logistical barriers for some women [105,106], persistent inequities in digital access underscore the need for screening strategies that are sensitive to both contextual risk and resource availability [107].

### 3.5. Evidence Base for a Risk-Stratified Perinatal Mental Health Screening Framework

Antenatal screening is increasingly conceptualized as a process of early risk identification, as clinically significant postpartum symptoms often emerge after delivery. Pregnancy-specific anxiety has been shown to represent a construct distinct from generalized anxiety and depressive symptomatology. Prospective longitudinal studies demonstrate that pregnancy-related anxiety follows distinct trajectories across gestation and is associated with later anxiety symptoms [108,109], supporting the inclusion of pregnancy-specific assessment tools such as the PRAQ-R2 during the second and third trimesters (Table 1).

Antenatal depressive symptoms also contribute meaningfully to postpartum risk. Longitudinal evidence indicates that depressive symptoms identified during pregnancy are associated with an increased likelihood of postpartum depression [110]. Importantly, screening timing influences predictive performance: second-trimester EPDS scores have demonstrated stronger predictive value for postpartum depressive symptoms than other antenatal timepoints [111], supporting mid-pregnancy as a high-yield window for risk stratification (Table 1).

Psychosocial adversity, particularly intimate partner violence (IPV), has emerged as a strong modifier of perinatal mental health trajectories. Cohort data indicate that IPV exposure is associated with greater symptom severity and slower recovery across the perinatal period [112], underscoring its relevance as a high-priority screening domain within antenatal risk assessment (Table 1).

Postpartum screening remains essential for the detection of clinically relevant symptoms. Diagnostic validation studies confirm the feasibility and accuracy of EPDS at routine postpartum visits when locally validated cutoffs are applied. In a rural and peri-urban Ugandan sample, EPDS demonstrated high sensitivity and specificity at six weeks postpartum [113], supporting its continued role as the core postpartum screening instrument (Table 1). However, symptom persistence may be influenced by relational factors beyond mood symptoms alone. Longitudinal evidence indicates that sexual dysfunction and poorer partnership quality are common in the perinatal period and may contribute to sustained psychological distress [71], supporting selective postpartum assessment of relational and sexual health among higher-risk women (Table 1).

**Table 1:** Key longitudinal, diagnostic, and relational evidence informing antenatal risk stratification and postpartum detection of perinatal depression and anxiety.

Screening Stage	Constructs & Instruments	Study (Year)	Design & Setting	Key Findings	Relevance to Framework
**Antenatal (2nd–3rd trimester)**	Pregnancy-specific anxiety (PRAQ-type); antenatal depressive symptoms (EPDS); psychosocial adversity (IPV)	Blackmore et al., 2016 [108]	Prospective longitudinal	Pregnancy-related anxiety is a **distinct construct** with clinical significance independent of general anxiety	Justifies PRAQ-R2 as an antenatal risk marker
		Mudra et al., 2020 [109]	Longitudinal cohort	PRAQ trajectories vary by parity and track later anxiety symptoms	Supports repeated antenatal assessment
		Tanuma-Takahashi et al., 2022 [111]	Prospective cohort	**Second-trimester EPDS cutoff 4/5** predicted postpartum EPDS ≥ 9 (Se 85.7%, Sp 77.1%)	Identifies mid-pregnancy as a high-yield screening window
		Hou et al., 2020 [112]	Cohort (trajectory analysis)	IPV associated with **greater severity and slower recovery** of perinatal depression	Positions IPV as a high-priority stratifier
		Luciano et al., 2022 [110]	Longitudinal “real-world” study	Antenatal EPDS predicts higher postpartum symptoms	Reinforces need for antenatal screening
**Postpartum (6–12 weeks)**	Core depression screen (EPDS); relational drivers (FSFI, partnership quality); IPV	Atuhaire et al., 2023 [113]	Diagnostic validation	EPDS ≥ 10 at 6 weeks postpartum: **Se 86.8%, Sp 92.1%** vs. MINI	Confirms EPDS feasibility and accuracy at routine postpartum visit
		Wallwiener et al., 2017 [71]	Longitudinal cohort	26–35% at risk of sexual dysfunction (FSFI < 26.55); low partnership quality and breastfeeding linked to poorer outcomes	Supports selective postpartum assessment of sexual/relational context
		Solomonov et al., 2025 [114]	Policy implementation study	Mandatory EPDS increased screening (1.0% → 14.2%); **17.1% linked to care**	Demonstrates impact of system-level implementation

Abbreviations: EPDS = Edinburgh Postnatal Depression Scale; FSFI = Female Sexual Function Index; IPV = intimate partner violence; MINI = Mini-International Neuropsychiatric Interview; PRAQ = Pregnancy-Related Anxiety Questionnaire.

Figure 2 synthesizes this evidence into a pragmatic, staged screening protocol integrating pregnancy-specific anxiety assessment, psychosocial risk stratification, and selective postpartum evaluation of relational domains for high-risk women.

Implementation-focused evidence highlights that screening effectiveness depends on system-level integration rather than instrument choice alone. High-income maternity care frameworks emphasize universal screening embedded within defined workflows and referral pathways (Kendig et al., 2017 [115]). Empirical implementation data from a large urban medical center demonstrated that mandatory EPDS screening substantially increased screening coverage and facilitated linkage to mental health services (Solomonov et al., 2025 [114]). In low-resource and rural settings, systematic reviews emphasize the importance of local validation, staff training, and referral capacity to ensure feasibility and sustainability (Gyimah et al., 2024 [116]). Together, these findings underscore that screening should be conceptualized as a care pathway rather than a standalone activity (Table 2).

**Table 2:** Operational lessons from high-income and low-resource maternity care programs relevant to protocol design.

Program/Study	Setting	Timing & Frequency	Instruments	Referral & Workflow	Key Implementation Outcomes
Kendig et al., 2017 (Consensus Bundle) [115]	High-income maternity systems	Prenatal and postpartum; repeated surveillance encouraged	EPDS/PHQ-9 ± anxiety tools; psychosocial risk	Integrated clinician-led screening with defined referral pathways	Provides system-level framework for effective screening
Solomonov et al., 2025 [114]	USA, urban academic center	Routine perinatal screening (policy-mandated)	Mandatory EPDS	Embedded clinic screening with mental health referral	Increased coverage; measurable linkage-to-care
Luciano et al., 2022 [110]	Italy, university hospital	Pregnancy and postpartum follow-up	EPDS	Screening embedded in routine obstetric care	Demonstrates feasibility of longitudinal screening
Atuhaire et al., 2023 [113]	Uganda, rural/peri-urban clinics	6-week postpartum visit	EPDS (local language)	Private clinic screening with referral	Validated local cutoff; high diagnostic accuracy
Gyimah et al., 2024 [116]	Sub-Saharan Africa primary care	Routine antenatal & postpartum visits	EPDS, PHQ-9 most supported	Emphasizes staff training and referral capacity	Highlights feasibility and documentation gaps

Abbreviations: EPDS = Edinburgh Postnatal Depression Scale; PHQ-9 = Patient Health Questionnaire-9.

To facilitate clinical implementation, Table 3 provides practical guidance on instrument selection, administration time, and workflow integration for the proposed screening framework. Instruments are categorized as “core” (universal screening: EPDS, PRAQ-R2, GAD-2) or “adjunct” (selective risk-stratified assessment: GAD-7, FSFI, RSES, MSPSS, IPV screening). Recognizing time constraints as a primary implementation barrier, particular attention is given to pragmatic sexual function screening, with three tiered options ranging from single-item questions (<1 min) to the full FSFI (5–7 min). All instruments are self-administered, with total screening burden ranging from 5–6 min for universal antenatal screening to 12–20 min for comprehensive postpartum assessment in high-risk women.

**Table 3:** Practical Implementation Guide: Screening Instruments for Risk-Stratified Perinatal Mental Health Assessment.

Instrument	Category	Timing	Items	Duration	Feasibility & Implementation Notes
CORE INSTRUMENTS (Universal Screening)					
EPDS	Core	2nd trimester, 3rd trimester, 6 weeks postpartum	10	2–3 min	Self-administered; widely validated; available in 50+ languages; can be completed in waiting room; minimal clinician time
PRAQ-R2	Core	2nd–3rd trimester only	10	2–3 min	Pregnancy-specific anxiety; self-administered; validates universal antenatal screening beyond depression alone
GAD-2	Core	6 weeks postpartum	2	<1 min	Ultra-brief anxiety screen; embedded in postpartum visit workflow
ENHANCED SCREENING (Selective for Elevated Scores: EPDS ≥ 13 or PRAQ-R2 ≥ 26)					
GAD-7	Adjunct	Antenatal if EPDS/PRAQ-R2 elevated	7	2 min	Self-administered; distinguishes generalized vs. pregnancy-specific anxiety
IPV screening	Adjunct	Antenatal if risk indicators present	3–5 *	1–2 min	Brief validated tools (HITS, E-HITS, or single-item); requires private setting; mandatory reporter considerations
MSPSS	Adjunct	Antenatal if high-risk; postpartum for moderate/high-risk	12	3–4 min	Assesses perceived partner, family, and friend support; self-administered
TARGETED POSTPARTUM ASSESSMENT (Selective for Moderate/High-Risk Women Only)					
FSFI (full)	Adjunct	6–12 weeks postpartum (high-risk only)	19	5–7 min	Full version: Comprehensive assessment if sexual concerns endorsed or depression persists; requires sexual activity in past 4 weeks
FSFI-6 (brief)	Adjunct	6 weeks postpartum (pragmatic option)	6	2–3 min	Brief alternative: Single items from each FSFI domain; screening efficiency; positive screen → full FSFI or clinical discussion
Sexual function screening items	Adjunct	6 weeks postpartum (maximum pragmatism)	1–2	<1 min	Single-item options: “Are you satisfied with your sexual relationship?” or “Has sexual function been a concern since delivery?” Positive → FSFI-6 or referral
RSES	Adjunct	6–12 weeks postpartum (moderate/high-risk)	10	2–3 min	Self-esteem assessment; self-administered; predicts symptom persistence

### 3.6. Antenatal Psychological Vulnerabilities

Pregnancy-specific anxiety and general pre-natal distress were found to be reliable predictors of adverse outcomes after delivery. Longitudinal cohort studies have shown that higher levels of pre-natal anxiety are associated with an increased likelihood of a follow-up of a depressive-like pattern and moderate to severe sexual dysfunction during the first year of postpartum. In a large cohort of first-time mothers (N = 646), psychosocial distress during pregnancy predicted the likelihood of high-risk sexual function trajectories measured at three, six and 12 months postpartum [92].

Additionally, dyadic longitudinal analyses highlighted the interpersonal context of postpartum adjustment by showing a strong correlation between postpartum depressive symptoms and sexual dysfunction and distress in both partners [93].

One of the biggest prenatal risk factors was body image issues. Research from the Norwegian Mother and Child Cohort Study (MoBa; N ≈ 39,915) showed that weight gain during pregnancy predicted depressive symptoms later on by affecting body image dissatisfaction. Moderated mediation was indicated by the significantly stronger mediation pathway among women with higher pre-pregnancy BMI (body mass index) [94].

Relatedly, perinatal self-objectification, operationalized as body surveillance, predicted higher prenatal depressive symptoms, greater postpartum body dissatisfaction, and impaired mother–infant bonding at 12 months postpartum, suggesting downstream consequences extending beyond maternal mood [95].

Relationship satisfaction and perceived partner support also functioned as key antenatal and early postpartum risk modifiers. Lower relationship satisfaction measured during pregnancy was prospectively associated with adverse sexual and depressive symptom trajectories postpartum [92]. Cross-sectional population data corroborated these findings, showing that higher relationship satisfaction was associated with substantially reduced odds of postpartum sexual dysfunction and sexual distress [96].

Finally, prior mental health history consistently predicted poorer postpartum outcomes. Women with pre-existing or antenatal depressive disorders exhibited lower sexual functioning across pregnancy and postpartum, with persistent dysfunction among those whose depressive symptoms continued through the first postpartum year.

Sexual function and sexual distress emerged as salient early postpartum markers. Across longitudinal studies, sexual function typically declined from pregnancy to approximately three months postpartum, followed by gradual recovery toward 12 months. Depressive symptoms measured in early postpartum predicted poorer sexual functioning and higher sexual distress at three months, although they did not consistently predict later recovery trajectories [93].

Cross-sectional evidence further demonstrated moderate inverse associations between sexual function and depressive symptoms. In a postpartum sample (N = 147), higher EPDS scores were associated with increased odds of sexual dysfunction, and FSFI scores were negatively correlated with depressive symptom severity [97].

Postpartum body image dissatisfaction also contributed to symptom persistence. Longitudinal population data showed that body image dissatisfaction often increased postpartum—particularly among women with higher BMI—and was associated with greater depressive symptom severity [98].

Across studies, body image and sexual satisfaction functioned as central mediators linking physiological, psychological, and relational factors to postpartum depression. The most robust mediation evidence supported pathways in which gestational weight gain influenced postpartum depressive symptoms indirectly through body image dissatisfaction, with stronger effects in women with higher pre-pregnancy BMI [94].

In the early postpartum period, dyadic longitudinal analyses also revealed that sexual satisfaction mediated relationships between body satisfaction and relationship satisfaction, emphasizing the dynamic interdependencies between relational and individual processes [98].

## 4. Implications for Practice and Policy

Regular screening for depression and anxiety should be a standard part of care during key stages of pregnancy and the postpartum period. This includes clear referral pathways and short psychosocial interventions that can be offered when symptoms are identified. Research consistently shows that common mental health disorders are more frequent during the perinatal period than at other times in a woman’s life [117,118]. Reflecting this, the U.S. Preventive Services Task Force recommends routine screening for depression and anxiety in both pregnant and postpartum women to ensure problems are recognized early and managed appropriately [119].

Creating simple and reliable routes for women to reach mental health support is equally important, especially for those in rural or underserved areas. Studies have pointed out that screening for maternal mental health problems remains patchy and inconsistent across many care systems, leaving an important gap in service delivery [120]. Expanding the use of telehealth could help bridge that gap, giving women the chance to receive timely psychological support without needing to travel long distances [121].

**Figure 3:**
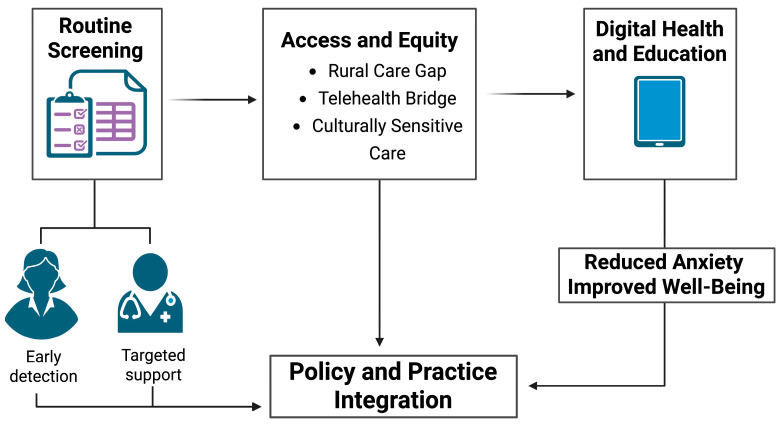
Integrated strategies to enhance perinatal mental health care.

Another crucial element is making sure these services are culturally sensitive and responsive to the needs of diverse populations. Women from lower socioeconomic backgrounds or minority communities often face additional barriers and are at a greater risk of depression both before and after giving birth [122]. Policies aimed at improving access to high-quality mental health care for these groups could have lasting benefits for both maternal and infant well-being (Figure 3).

In recent years, attention has also turned to how digital habits—particularly smartphone and social media use—can influence anxiety levels during and after pregnancy. Excessive exposure to distressing online content or continuous news updates has been linked to higher levels of anxiety [123,124,125,126]. For this reason, digital health programs such as mHealth or telehealth should not only increase access to care but also include advice on healthy media use, encouraging moderation and helping women recognize when online engagement may be contributing to stress [127].

Tools like the EPDS remain essential for identifying depression and related anxiety symptoms efficiently [128]. Beyond screening, interventions should be designed to reflect the complex and interconnected nature of perinatal mental health, particularly as modern lifestyles become more digitally driven [59].

## 5. Limitation

This narrative evidence synthesis should be interpreted in light of several methodological limitations.

First, the review design was narrative rather than systematic. Although the search strategy was structured and conducted across multiple databases, the approach does not provide the same reproducibility, auditability, or exhaustiveness as a preregistered systematic review. As a result, relevant studies may have been missed, and study selection may be more vulnerable to inadvertent selection bias.

Second, no formal, study-level risk-of-bias appraisal (Newcastle–Ottawa Scale for observational studies, RoB 2 for trials, AMSTAR 2 for systematic reviews) was applied across included evidence. Instead, interpretive weight was guided qualitatively by design, sample size, measurement validity, timing clarity, and confounding adjustment. This limits the ability to grade certainty of evidence and may overemphasize findings from studies with unmeasured bias.

A related challenge concerns causal inference and directionality. The evidence base is predominantly observational, and the relationship between sexual dysfunction and depression/anxiety is almost certainly bidirectional: mood symptoms may reduce desire, arousal, and satisfaction through both psychological (e.g., anhedonia, negative cognitions) and physiological pathways (e.g., medication side effects, fatigue), while persistent sexual difficulties may intensify relational strain, erode partner support, and contribute to ongoing psychological distress. Few studies employed designs or analytic methods (e.g., cross-lagged panel models, instrumental variable approaches) capable of disentangling these reciprocal effects, and residual confounding by unmeasured factors (e.g., baseline mental health history, relationship quality prior to pregnancy, chronic pain, breastfeeding practices) remains plausible.

Crucially, very few studies directly tested the incremental predictive value of adding sexual function (FSFI) or self-esteem/body image (RSES) assessments to standard depression screening (EPDS) in terms of improved sensitivity, specificity, positive predictive value, or clinical outcomes. The screening framework we propose is therefore inferential, synthesizing evidence that (i) sexual dysfunction and low self-esteem are associated with perinatal mood/anxiety symptoms, (ii) these associations persist after adjusting for baseline symptoms in some studies, and (iii) these domains are feasible to assess. However, prospective validation studies comparing single-instrument vs. multi-domain screening protocols—ideally with randomization to screening strategy and long-term follow-up of maternal and infant outcomes—are needed to establish whether the proposed approach improves detection, linkage-to-care, or clinical outcomes relative to standard practice.

Third, heterogeneity across the evidence base constrains inference. Included studies varied substantially in (i) perinatal timing windows (trimester-specific vs. broad antenatal; 6 weeks vs. 12 months postpartum), (ii) populations and settings (high-income vs. low-resource; rural vs. urban), and (iii) instruments and cutoffs (EPDS, PHQ-9, GAD-7, PRAQ/PRAQ-R2, FSFI, RSES, MSPSS, and diagnostic interviews). Such variability impedes direct comparison and precluded quantitative pooling; consequently, the review emphasizes convergent patterns rather than effect-size precision.

Fourth, much of the underpinning evidence is observational and frequently cross-sectional, which restricts causal interpretation. Associations between sexual function, self-esteem/body image, psychosocial adversity, and perinatal mood/anxiety symptoms may reflect bidirectional effects and residual confounding (e.g., baseline mental health history, relationship quality, breastfeeding, obstetric complications, pain, sleep disruption). Even in longitudinal studies, incomplete adjustment—particularly for antenatal depression/anxiety when examining pregnancy-specific anxiety or postpartum relational factors—may inflate or obscure independent effects.

Fifth, measurement limitations may affect comparability and clinical translation. Self-report instruments are vulnerable to reporting bias and social desirability effects, especially for sexual functioning and IPV. FSFI applicability can vary across postpartum physiological states (dyspareunia, lactational hypoestrogenism) and cultural contexts; similarly, locally validated EPDS cutoffs differ by language and setting, limiting generalizability of single threshold recommendations.

Sixth, the screening-framework implications presented are inferential. While the synthesis supports the plausibility of staged, risk-stratified screening that integrates pregnancy-specific anxiety antenatally and relational–psychological markers postpartum, few studies directly test whether adding FSFI/RSES (or similar measures) improves predictive performance, clinical outcomes, equity, or linkage-to-care compared with standard screening alone. Implementation claims should therefore be read as practice-oriented hypotheses grounded in converging evidence rather than definitive protocol validation.

## 6. Conclusions

Perinatal depression and anxiety are common and under-detected when screening focuses on depressive symptoms alone. This review supports a staged, risk-stratified model in which antenatal screening integrates pregnancy-specific anxiety and psychosocial risk, and postpartum assessment selectively incorporates sexual function and self-esteem domains for symptomatic or high-risk women. Because direct evidence on the incremental performance of combined screening is limited, prospective validation and implementation studies are needed to determine whether this approach improves detection, referral, and outcomes relative to standard practice.

## Data Availability

No new data were created or analyzed in this study. Data sharing is not applicable to this article.

## References

[1] Alizadeh S., Riazi H., Alavi-Majd H., Ozgoli G. (2022). Prevalence of Female Sexual Dysfunction during Pregnancy in Eastern Mediterranean Regional Office Countries (EMRO): A Systematic Review and Meta-Analysis. J. Matern. Fetal Neonatal Med..

[2] Pabon S., Parpinelli M.A., Narvaez M.B., Charles C.M., Guida J.P., Escobar M.F., Cecatti J.G., Costa M.L. (2020). Overall Maternal Morbidity during Pregnancy Identified with the WHO-WOICE Instrument. BioMed Res. Int..

[3] Chen S., Luo T., Huang L., Zhou W., Luo J. (2022). Relationships between Sexual Function, Mental Health, and Quality of Life of Female Patients with Pulmonary Arterial Hypertension Associated with Congenital Heart Disease. Pulm. Circ..

[4] Becker M., Weinberger T., Chandy A., Schmukler S. (2016). Depression During Pregnancy and Postpartum. Curr. Psychiatry Rep..

[5] Afulani P.A., Ongeri L., Kinyua J., Temmerman M., Mendes W.B., Weiss S.J. (2021). Psychological and Physiological Stress and Burnout among Maternity Providers in a Rural County in Kenya: Individual and Situational Predictors. BMC Public Health.

[6] Hou Q., Li S., Jiang C., Huang Y., Huang L., Ye J., Pan Z., Teng T., Wang Q., Jiang Y. (2018). The Associations between Maternal Lifestyles and Antenatal Stress and Anxiety in Chinese Pregnant Women: A Cross-Sectional Study. Sci. Rep..

[7] Andreucci C.B., Filippi V., Cecatti J.G. (2021). Women’s Well-Being and Functioning after Evidence-Based Antenatal Care: A Protocol for a Systematic Review of Intervention Studies. BMJ Open.

[8] Effati-Daryani F., Jahanfar S., Mohammadi A., Zarei S., Mirghafourvand M. (2021). The Relationship between Sexual Function and Mental Health in Iranian Pregnant Women during the COVID-19 Pandemic. BMC Pregnancy Childbirth.

[9] Biresaw M.S., Takelle G.M., Gebeyehu E.T. (2022). Perceived Stress and Associated Factors among Pregnant Women during COVID-19 Pandemic Period in Northwest Ethiopia, 2020: A Cross-Sectional Study. BMJ Open.

[10] Paddy A., Asamoah-Gyimah K., Nkyi A., Paddy A., Asamoah-Gyimah K., Nkyi A. (2021). Psychosocial Determinants of Postpartum Depression and Maternal Well-Being among Postnatal Women. Open J. Psychiatry.

[11] Liu X., Wang S., Wang G. (2022). Prevalence and Risk Factors of Postpartum Depression in Women: A Systematic Review and Meta-Analysis. J. Clin. Nurs..

[12] O’Malley D., Higgins A., Begley C., Daly D., Smith V. (2018). Prevalence of and Risk Factors Associated with Sexual Health Issues in Primiparous Women at 6 and 12 Months Postpartum; a Longitudinal Prospective Cohort Study (the MAMMI Study). BMC Pregnancy Childbirth.

[13] Hababa H., Sine H., Boukrim M., Lafdili L., Assarag B. (2023). The Relationship Between Mental Health and Sexual Function in Moroccan Pregnant Women. Ann. Public Health Res..

[14] Yin X., Sun N., Jiang N., Xu X., Gan Y., Zhang J., Qiu L., Yang C., Shi X., Chang J. (2021). Prevalence and Associated Factors of Antenatal Depression: Systematic Reviews and Meta-Analyses. Clin. Psychol. Rev..

[15] Chauhan A., Potdar J. (2022). Maternal Mental Health During Pregnancy: A Critical Review. Cureus.

[16] Bedaso A., Adams J., Peng W., Sibbritt D. (2021). The Relationship between Social Support and Mental Health Problems during Pregnancy: A Systematic Review and Meta-Analysis. Reprod. Health.

[17] Lobel M., Ibrahim S.M. (2018). Emotions and Mental Health During Pregnancy and Postpartum. Women’s Reprod. Health.

[18] Haiman M., Cubbin C. (2023). Impact of Geography and Rurality on Preconception Health Status in the United States. Prev. Chronic Dis..

[19] Diggikar S., Galis R., Nagesh K., Pandita A., Ognean M.L., Rüdiger M., Mazela J., Kramer B.W. (2024). Surfactant Therapy—The Conundrum of Which Infant Should Be given, When, Which Drug in What Dose via Which Route of Administration?. Semin. Fetal Neonatal Med..

[20] Hung P., Henning-Smith C.E., Casey M.M., Kozhimannil K.B. (2017). Access to Obstetric Services in Rural Counties Still Declining, with 9 Percent Losing Services, 2004–2014. Health Aff..

[21] Fontenot J., Brigance C., Lucas R., Stoneburner A. (2024). Navigating Geographical Disparities: Access to Obstetric Hospitals in Maternity Care Deserts and Across the United States. BMC Pregnancy Childbirth.

[22] Souaibou M., Sandie A.B., Barros A.J.D., Dzossa A.D., Sidze E. (2025). Inequalities in Effective Coverage of the Maternal Healthcare Continuum in Cameroon: A Cascade Analysis from Service Contact to Input-Adjusted Coverage. BMC Health Serv. Res..

[23] Igunza K.A., Ramakrishna N., Madewell Z.J., Akelo V., Muttai H., Mugah C., Mitei P.K., Ogbuanu I.U., Bassey I.-A., Kaluma E. (2025). Elucidating Delays in Illness Recognition, Healthcare Seeking, and Healthcare Provision for Stillbirths and Neonatal Deaths in Seven Low- and Middle-Income Countries. medRxiv.

[24] Watson H., Harrop D., Walton E., Young A.W., Soltani H. (2019). A Systematic Review of Ethnic Minority Women’s Experiences of Perinatal Mental Health Conditions and Services in Europe. PLoS ONE.

[25] Lee J., Howard K.J., Leong C., Grigsby T.J., Howard J.T. (2024). Delayed Care during Pregnancy and Postpartum Linked to Poor Maternal Mental Health: Evidence in the United States. J. Reprod. Infant. Psychol..

[26] Kaza P.S. (2025). Unveiling the Challenges and Solutions: A Scoping Review of Maternal Healthcare Access in Rural Georgia. Cureus.

[27] Garcia K.K., Hunter S.K. (2022). Proposed Solutions for Improving Maternal Health Care in Rural America. Clin. Obstet. Gynecol..

[28] Dahab R., Sakellariou D. (2020). Barriers to Accessing Maternal Care in Low Income Countries in Africa: A Systematic Review. Int. J. Environ. Res. Public Health.

[29] Banke-Thomas A., Avoka C.K., Gwacham-Anisiobi U., Omololu O., Balogun M., Wright K., Fasesin T.T., Olusi A., Afolabi B.B., Ameh C. (2022). Travel of Pregnant Women in Emergency Situations to Hospital and Maternal Mortality in Lagos, Nigeria: A Retrospective Cohort Study. BMJ Glob. Health.

[30] Woodward R., Mazure E.S., Belden C.M., Denslow S., Fromewick J., Dixon S., Gist W., Sullivan M.H. (2023). Association of Prenatal Stress with Distance to Delivery for Pregnant Women in Western North Carolina. Midwifery.

[31] Mathews E., McNeill L., Cooper M., Briley A. (2024). Lost in Transition: Perspectives from Women and Their Families Living in Rural Australia on Relocation for Specialist Maternal and Neonatal Care. Women Birth.

[32] Guttikonda A., Shajan A.M., Hephzibah A., Jones A.S., Susanna J., Neethu S., Poornima S., Jala S.M., Arputharaj D., John D. (2019). Perceived Stigma Regarding Mental Illnesses among Rural Adults in Vellore, Tamil Nadu, South India. Indian J. Psychol. Med..

[33] Chebet T. (2024). Mental Health Awareness and Stigma in Rural vs. Urban Communities. Int. J. Humanit. Soc. Sci..

[34] Thi L.M., Manzano A., Ha B.T.T., Vui L.T., Quynh-Chi N.T., Duong D.T.T., Lakin K., Kane S., Mirzoev T., Trang D.T.H. (2024). Mental Health Stigma and Health-Seeking Behaviors amongst Pregnant Women in Vietnam: A Mixed-Method Realist Study. Int. J. Equity Health.

[35] Craemer K.A., Garland C.E., Sayah L., Duffecy J., Geller S.E., Maki P.M. (2023). Perinatal Mental Health in Low-Income Urban and Rural Patients: The Importance of Screening for Comorbidities. Gen. Hosp. Psychiatry.

[36] Ginja S., Jackson K., Newham J.J., Henderson E.J., Smart D., Lingam R. (2020). Rural-Urban Differences in the Mental Health of Perinatal Women: A UK-Based Cross-Sectional Study. BMC Pregnancy Childbirth.

[37] Waqas A., Raza N., Lodhi H.W., Muhammad Z., Jamal M., Rehman A. (2015). Psychosocial Factors of Antenatal Anxiety and Depression in Pakistan: Is Social Support a Mediator?. PLoS ONE.

[38] Shafaie F.S., Mirghafourvand M., Rahmati M., Nouri P., Bagherinia M. (2018). Association between Psychological Status with Perceived Social Support in Pregnant Women Referring to Tabriz Health Centers. J. Matern. Fetal Neonatal Med..

[39] Mohammed L.A., Negesse Simegn Y., Liyew A.D., Gelaw T., Wossen A., Chanyalew L., Endris S., Abebaw N., Desalegn S.Y. (2025). Factors Associated with Pregnancy-Related Anxiety among Pregnant Women Attending Antenatal Care at Public Health Institutions in Dessie Town, Northeast Ethiopia, 2023: An Institution-Based Cross-Sectional Study. BMJ Open.

[40] Redinger S., Norris S.A., Pearson R.M., Richter L., Rochat T. (2018). First Trimester Antenatal Depression and Anxiety: Prevalence and Associated Factors in an Urban Population in Soweto, South Africa. J. Dev. Orig. Health Dis..

[41] Khademi K., Kaveh M.H. (2024). Social Support as a Coping Resource for Psychosocial Conditions in Postpartum Period: A Systematic Review and Logic Framework. BMC Psychol..

[42] Sangsawang B., Deoisres W., Hengudomsub P., Sangsawang N. (2022). Effectiveness of Psychosocial Support Provided by Midwives and Family on Preventing Postpartum Depression among First-Time Adolescent Mothers at 3-Month Follow-up: A Randomised Controlled Trial. J. Clin. Nurs..

[43] Erbil N., Akin O. (2024). The Effect of Perceived Social Support on Pregnancy Stress: A Descriptive and Cross-Sectional Study. Int. J. Caring Sci..

[44] Kaydırak M.M., Balkan E., Bacak N., Kızoglu F. (2024). Perceived Social Support and Depression, Anxiety and Stress in Pregnant Women Diagnosed with Foetal Anomaly. J. Adv. Nurs..

[45] Arora I.K., Gehlawat P., Gupta T., Sharma C., Suthar N., Singh P., Goel A.D. (2025). Association of Pregnancy-Related Anxiety with Perceived Social Support: An Observational Study Among Third-Trimester Antenatal Women. Prim. Care Companion CNS Disord..

[46] Martin R.C., Brock R. (2023). The Importance of High Quality Partner Support for Reducing Stress During Pregnancy and Postpartum Bonding Impairments. Arch. Womens Ment. Health.

[47] Antoniou E., Tzanoulinou M.-D., Stamoulou P., Orovou E. (2022). The Important Role of Partner Support in Women’s Mental Disorders During the Perinatal Period. A Literature Review. Maedica J. Clin. Med..

[48] De Waal N., Boekhorst M.G.B.M., Nyklíček I., Pop V.J.M. (2023). Maternal-Infant Bonding and Partner Support during Pregnancy and Postpartum: Associations with Early Child Social-Emotional Development. Infant. Behav. Dev..

[49] Wang Y.-N., Yuan Z.-J., Leng W.-C., Xia L.-Y., Wang R.-X., Li Z.-Z., Zhou Y.-J., Zhang X.-Y. (2021). Role of Perceived Family Support in Psychological Distress for Pregnant Women during the COVID-19 Pandemic. World J. Psychiatry.

[50] Pilkington P.D., Whelan T.A., Milne L.C. (2016). Maternal Crying and Postpartum Distress: The Moderating Role of Partner Support. J. Reprod. Infant. Psychol..

[51] Khanna T., Patel R., Akhtar F., Mehra S. (2023). Relationship between Partner Support and Psychological Distress among Young Women during Pregnancy: A Mixed-Method Study from a Low- and Middle-Income Country. J. Affect. Disord. Rep..

[52] Woldetensay Y.K., Belachew T., Biesalski H.K., Ghosh S., Lacruz M.E., Scherbaum V., Kantelhardt E.J. (2018). The Role of Nutrition, Intimate Partner Violence and Social Support in Prenatal Depressive Symptoms in Rural Ethiopia: Community Based Birth Cohort Study. BMC Pregnancy Childbirth.

[53] Agarwal S., Prasad R., Mantri S., Chandrakar R., Gupta S., Babhulkar V., Srivastav S., Jaiswal A., Wanjari M.B. (2023). A Comprehensive Review of Intimate Partner Violence During Pregnancy and Its Adverse Effects on Maternal and Fetal Health. Cureus.

[54] Bacchus L.J., Ranganathan M., Watts C., Devries K. (2018). Recent Intimate Partner Violence against Women and Health: A Systematic Review and Meta-Analysis of Cohort Studies. BMJ Open.

[55] Halim N., Beard J., Mesic A., Patel A., Henderson D., Hibberd P. (2018). Intimate Partner Violence during Pregnancy and Perinatal Mental Disorders in Low and Lower Middle Income Countries: A Systematic Review of Literature, 1990–2017. Clin. Psychol. Rev..

[56] Rogathi J.J., Manongi R., Mushi D., Rasch V., Sigalla G.N., Gammeltoft T., Meyrowitsch D.W. (2017). Postpartum Depression among Women Who Have Experienced Intimate Partner Violence: A Prospective Cohort Study at Moshi, Tanzania. J. Affect. Disord..

[57] Çankaya S. (2020). The Effect of Psychosocial Risk Factors on Postpartum Depression in Antenatal Period: A Prospective Study. Arch. Psychiatr. Nurs..

[58] Necho M., Belete A., Zenebe Y. (2020). The Association of Intimate Partner Violence with Postpartum Depression in Women during Their First Month Period of Giving Delivery in Health Centers at Dessie Town, 2019. Ann. Gen. Psychiatry.

[59] Biaggi A., Conroy S., Pawlby S., Pariante C.M. (2016). Identifying the Women at Risk of Antenatal Anxiety and Depression: A Systematic Review. J. Affect. Disord..

[60] Suh E.Y., Ma P., Dunaway L.F., Theall K.P. (2016). Pregnancy Intention and Post-Partum Depressive Affect in Louisiana Pregnancy Risk Assessment Monitoring System. Matern. Child. Health J..

[61] Faisal-Cury A., Menezes P.R., Quayle J., Matijasevich A. (2017). Unplanned Pregnancy and Risk of Maternal Depression: Secondary Data Analysis from a Prospective Pregnancy Cohort. Psychol. Health Med..

[62] Zahid N., Blebu B., Felder J., McCulloch C.E., Chambers B.D., Curry V.C., Carraway K., León-Martínez D., Coleman-Phox K., Kuppermann M. (2025). Economic Insecurities and Mental Health Among Low-Income Pregnant People in the Central Valley Region of California. Women’s Health Issues.

[63] Katz J., Crean H.F., Cerulli C., Poleshuck E.L. (2018). Material Hardship and Mental Health Symptoms Among a Predominantly Low Income Sample of Pregnant Women Seeking Prenatal Care. Matern. Child Health J..

[64] Barton K., Redshaw M., Quigley M.A., Carson C. (2017). Unplanned Pregnancy and Subsequent Psychological Distress in Partnered Women: A Cross-Sectional Study of the Role of Relationship Quality and Wider Social Support. BMC Pregnancy Childbirth.

[65] Bogdan I., Turliuc M.N., Candel O.S. (2022). Transition to Parenthood and Marital Satisfaction: A Meta-Analysis. Front. Psychol..

[66] Chesli S.R., Bostani Khalesi Z., Chenari S.S. (2024). The Role of Sexual Self-Esteem, Sexual Desire, and Sexual Assertiveness in the Female Sexual Function. Psicol. Reflex. Crit..

[67] Costas T., Gomes-Ferreira M., Gomes-Ferreira M., Olivas-Menayo J. (2023). Physiological and Hormonal Changes During Pregnancy. Post-Maternity Body Changes: Obstetric Fundamentals and Surgical Reshaping.

[68] Monteiro M.N., Lucena E.E.d.S., Cabral P.U., Queiroz Filho J., Queiroz J., Gonçalves A.K. (2016). Prevalence of Sexual Dysfunction among Expectant Women. Rev. Bras. Ginecol. Obs..

[69] Gutzeit O., Levy G., Lowenstein L. (2019). Postpartum Female Sexual Function: Risk Factors for Postpartum Sexual Dysfunction. Sex. Med..

[70] Rosen N.O., Pukall C. (2016). Comparing the Prevalence, Risk Factors, and Repercussions of Postpartum Genito-Pelvic Pain and Dyspareunia. Sex. Med. Rev..

[71] Wallwiener S., Müller M., Doster A., Kuon R.J., Plewniok K., Feller S., Wallwiener M., Reck C., Matthies L.M., Wallwiener C. (2017). Sexual Activity and Sexual Dysfunction of Women in the Perinatal Period: A Longitudinal Study. Arch. Gynecol. Obs..

[72] Erbil N. (2018). Sexual Function of Pregnant Women in the Third Trimester. Alex. J. Med..

[73] Gałązka I., Drosdzol-Cop A., Naworska B., Czajkowska M., Skrzypulec-Plinta V. (2015). Changes in the Sexual Function during Pregnancy. J. Sex. Med..

[74] Cassis C., Mukhopadhyay S., Morris E., Giarenis I. (2021). What Happens to Female Sexual Function during Pregnancy?. Eur. J. Obstet. Gynecol. Reprod. Biol..

[75] Eskitzis P., Michou V., Arampatzi C., Tsakiridis I., Papoutsis D. (2025). Emotional Disorders, Risk Factors, and Correlations of Post-Partum Depression and Post-Traumatic Stress Disorder with Sexual Function During Post-Partum Period. Diagnostics.

[76] Basson R., Gilks T. (2018). Women’s Sexual Dysfunction Associated with Psychiatric Disorders and Their Treatment. Women’s Health.

[77] Weber E., Hopwood C.J., Denissen J.J.A., Bleidorn W. (2024). Self-Esteem and Sexual Experiences. Pers. Soc. Psychol. Bull..

[78] Linde K., Lehnig F., Nagl M., Stepan H., Kersting A. (2022). Course and Prediction of Body Image Dissatisfaction during Pregnancy: A Prospective Study. BMC Pregnancy Childbirth.

[79] Hasani S., Aung E., Mirghafourvand M. (2021). Low Self-Esteem Is Related to Depression and Anxiety during Recovery from an Ectopic Pregnancy. BMC Women’s Health.

[80] Saqib K., Khan A.F., Butt Z.A. (2021). Machine Learning Methods for Predicting Postpartum Depression: Scoping Review. JMIR Ment. Health.

[81] Alkhateeb M., Nayeem A., Ahmed A., Alsahli M., Sheikh J., Abd-Alrazaq A. (2026). AI for Detecting and Predicting Postpartum Depression: Scoping Review. J. Med. Internet Res..

[82] Xia J., Chen C., Lu X., Zhang T., Wang T., Wang Q., Zhou Q. (2025). Artificial Intelligence-Oriented Predictive Model for the Risk of Postpartum Depression: A Systematic Review. Front. Public Health.

[83] Wakefield C., Frasch M.G. (2022). Identifying Patients Requiring Treatment for Depression in the Postpartum Period from Common Electronic Medical Record Data Available Antepartum Using Machine Learning. medRxiv.

[84] Ma Z., Horvath M., Stamilio D.M., Sekyere K., Gurcan M.N. (2025). Building a Machine Learning Model to Predict Postpartum Depression from Electronic Health Records in a Tertiary Care Setting. J. Clin. Med..

[85] Balsam D., Bounds D.T., Rahmani A.M., Nyamathi A. (2023). Evaluating the Impact of an App-Delivered Mindfulness Meditation Program to Reduce Stress and Anxiety During Pregnancy: Pilot Longitudinal Study. JMIR Pediatr. Parent..

[86] Sun Y., Li Y., Wang J., Chen Q., Bazzano A.N., Cao F. (2021). Effectiveness of Smartphone-Based Mindfulness Training on Maternal Perinatal Depression: Randomized Controlled Trial. J. Med. Internet Res..

[87] Rostamikia Z., Khajavian N., Rahmani Bilandi R., Askari F. (2023). The Effect of Educational Intervention with Mobile Health Technology on COVID-19 Induced Stress among Pregnant Women: A Randomized Controlled Trial. J. Midwifery Reprod. Health.

[88] Mina I., Hadis B., Saeedeh A., Saeed G., Beheshti N.M., Sara M. (2023). Effect of Tele-Medicine on Health Anxiety and Pregnancy-Related Anxiety in Pregnant Women during the COVID-19 Epidemic in Iran. Iran. J. Nurs. Midwifery Res..

[89] Li J., Silvera-Tawil D., Varnfield M., Hussain M.S., Math V. (2021). Users’ Perceptions Toward mHealth Technologies for Health and Well-Being Monitoring in Pregnancy Care: Qualitative Interview Study. JMIR Form. Res..

[90] Galis R., Trif P., Mudura D., Murvai R., Daina L.G., Szasz F., Negrini R., Hatos A., Gyarmati B.F., Daly M.C. (2024). Preterm Birth and Stillbirth during COVID-19 Pandemic in Bihor County/Romania. Front. Reprod. Health.

[91] Ogur Y.S., Yazıcı E., Yuvacı H.U., Ogur N.B., Köse E., Yazıcı A.B. (2023). Development of a Mobile Monitoring Program for Anxiety and Depression in Pregnancy and Evaluation of 3-Month Results. Sage J..

[92] Dawson S.J., Vaillancourt-Morel M.-P., Pierce M., Rosen N.O. (2020). Biopsychosocial Predictors of Trajectories of Postpartum Sexual Function in First-Time Mothers. Health Psychol..

[93] Dawson S.J., Leonhardt N.D., Impett E.A., Rosen N.O. (2021). Associations Between Postpartum Depressive Symptoms and Couples’ Sexual Function and Sexual Distress Trajectories Across the Transition to Parenthood. Ann. Behav. Med..

[94] Han S.-Y., Brewis A.A., Wutich A. (2016). Body Image Mediates the Depressive Effects of Weight Gain in New Mothers, Particularly for Women Already Obese: Evidence from the Norwegian Mother and Child Cohort Study. BMC Public Health.

[95] Laifer L.M., Maras O.R., Sáez G., Gervais S.J., Brock R.L. (2023). Self-Objectification During the Perinatal Period: The Role of Body Surveillance in Maternal and Infant Wellbeing. Sex. Roles.

[96] Wassenaar E., Lont F., Verhoeven C., Henrichs J., Titulaer L., Warmelink J., Geerts C. (2024). Sexual Health after Childbirth in Dutch Women: Prevalence, Associated Factors and Perceived Need for Information: A Cross-Sectional Study. BMC Pregnancy Childbirth.

[97] Chivers M.L., Pittini R., Grigoriadis S., Villegas L., Ross L.E. (2011). The Relationship Between Sexual Functioning and Depressive Symptomatology in Postpartum Women: A Pilot Study. J. Sex. Med..

[98] Linde K., Lehnig F., Treml J., Nagl M., Stepan H., Kersting A. (2024). The Trajectory of Body Image Dissatisfaction during Pregnancy and Postpartum and Its Relationship to Body-Mass-Index. PLoS ONE.

[99] O’Hara M.W., Wisner K.L. (2014). Perinatal Mental Illness: Definition, Description and Aetiology. Best. Pract. Res. Clin. Obstet. Gynaecol..

[100] Mateus V., Cruz S., Costa R., Mesquita A., Christoforou A., Wilson C.A., Vousoura E., Dikmen-Yildiz P., Bina R., Domínguez-Salas S. (2022). Rates of Depressive and Anxiety Symptoms in the Perinatal Period During the COVID-19 Pandemic: Comparisons Between Countries and with Pre-Pandemic Data. J. Affect. Disord..

[101] Caropreso L., Saliba S., Hasegawa L., Lawrence J., Davey C.J., Frey B.N. (2020). Quality Assurance Assessment of a Specialized Perinatal Mental Health Clinic. BMC Pregnancy Childbirth.

[102] Burton L., Milad F., Janke R., Rush K.L. (2024). The Landscape of Health Technology for Equity Deserving Groups in Rural Communities: A Systematic Review. Community Health Equity Res. Policy.

[103] Weinstein J., Bozkurt B., Aijaz M., Cilenti D., Khairat S., Shea C.M., Planey A.M. (2025). Community-Level Internet Connectivity and Utilization of Maternal Telehealth. Telemed. e-Health.

[104] Falah-Hassani K., Shiri R., Dennis C. (2017). The Prevalence of Antenatal and Postnatal Co-Morbid Anxiety and Depression: A Meta-Analysis. Psychol. Med..

[105] Dixon-Shambley K., Gabbe P.T. (2021). Using Telehealth Approaches to Address Social Determinants of Health and Improve Pregnancy and Postpartum Outcomes. Clin. Obstet. Gynecol..

[106] Adeogun A., Faezipour M. (2025). Assessing the Impact of Telemedicine on Patient Satisfaction Before and During the COVID-19 Pandemic. Healthcare.

[107] Patel S., Sun E., Reinhardt A., Geevarghese S., He S., Gazmararian J.A. (2022). Social Determinants of Digital Health Adoption: Pilot Cross-Sectional Survey. JMIR Form. Res..

[108] Blackmore E.R., Gustafsson H., Gilchrist M., Wyman C., G O’Connor T. (2016). Pregnancy-Related Anxiety: Evidence of Distinct Clinical Significance from a Prospective Longitudinal Study. J. Affect. Disord..

[109] Mudra S., Göbel A., Barkmann C., Goletzke J., Hecher K., Schulte-Markwort M., Diemert A., Arck P. (2020). The Longitudinal Course of Pregnancy-Related Anxiety in Parous and Nulliparous Women and Its Association with Symptoms of Social and Generalized Anxiety. J. Affect. Disord..

[110] Luciano M., Di Vincenzo M., Brandi C., Tretola L., Toricco R., Perris F., Volpicelli A., Torella M., La Verde M., Fiorillo A. (2022). Does Antenatal Depression Predict Post-Partum Depression and Obstetric Complications? Results from a Longitudinal, Long-Term, Real-World Study. Front. Psychiatry.

[111] Tanuma-Takahashi A., Tanemoto T., Nagata C., Yokomizo R., Konishi A., Takehara K., Ishikawa T., Yanaihara N., Samura O., Okamoto A. (2022). Antenatal Screening Timeline and Cutoff Scores of the Edinburgh Postnatal Depression Scale for Predicting Postpartum Depressive Symptoms in Healthy Women: A Prospective Cohort Study. BMC Pregnancy Childbirth.

[112] Hou F., Zhang X., Cerulli C., He W., Mo Y., Gong W. (2020). The Impact of Intimate Partner Violence on the Trajectory of Perinatal Depression: A Cohort Study in a Chinese Sample. Epidemiol. Psychiatr. Sci..

[113] Atuhaire C., Rukundo G.Z., Atwine D., Brennaman L., Nambozi G., Cumber S.N., Ngonzi J. (2021). Prevalence of Postpartum Depression and Associated Factors among Women in Mbarara and Rwampara Districts of South-Western Uganda. BMC Pregnancy Childbirth.

[114] Solomonov N., Kerchner D., Dai Y., Kwon M., Callaghan D.G., Schier M.M., Zhang Y., Osborne L.M., Benda N.C. (2025). Prevalence and Trajectories of Perinatal Anxiety and Depression in a Large Urban Medical Center. JAMA Netw. Open.

[115] Kendig S., Keats J.P., Hoffman M.C., Kay L.B., Miller E.S., Simas T.A.M., Frieder A., Hackley B., Indman P., Raines C. (2017). Consensus Bundle on Maternal Mental Health: Perinatal Depression and Anxiety. J. Midwifery Womens Health.

[116] Gyimah L., Agyepong I.A., Owiredu D., Awini E., Yevoo L.L., Ashinyo M.E., Aye S.G.E.V., Abbas S., Cronin de Chavez A., Mirzoev T. (2024). Tools for Screening Maternal Mental Health Conditions in Primary Care Settings in Sub-Saharan Africa: Systematic Review. Front. Public Health.

[117] Gelaye B., Rondón M.B., Araya R., Williams M.A. (2016). Epidemiology of Maternal Depression, Risk Factors, and Child Outcomes in Low-Income and Middle-Income Countries. Lancet Psychiatry.

[118] Woody C., Ferrari A.J., Siskind D., Whiteford H., Harris M. (2017). A Systematic Review and Meta-Regression of the Prevalence and Incidence of Perinatal Depression. J. Affect. Disord..

[119] Rafferty J., Mattson G., Earls M.F., Yogman M.W. (2018). Incorporating Recognition and Management of Perinatal Depression into Pediatric Practice. Pediatrics.

[120] Blackmore R., Boyle J., Gray K.M., Willey S., Highet N., Gibson-Helm M. (2021). Introducing and Integrating Perinatal Mental Health Screening: Development of an Equity-Informed Evidence-Based Approach. Health Expect..

[121] Baron E., Hanlon C., Mall S., Honikman S., Breuer E., Kathree T., Luitel N.P., Nakku J., Lund C., Medhin G. (2016). Maternal Mental Health in Primary Care in Five Low- And Middle-Income Countries: A Situational Analysis. BMC Health Serv. Res..

[122] Grote N.K., Katon W., Russo J., Lohr M.J., Curran M., Galvin E., Carson K. (2015). Collaborative Care for Perinatal Depression in Socioeconomically Disadvantaged Women: A Randomized Trial. Depress. Anxiety.

[123] Islam S., Sujan S.H., Tasnim R., Mohona R.A., Ferdous M.Z., Kamruzzaman S., Toma T.Y., Sakib M.N., Pinky K.N., Islam M.R. (2021). Problematic Smartphone and Social Media Use Among Bangladeshi College and University Students Amid COVID-19: The Role of Psychological Well-Being and Pandemic Related Factors. Front. Psychiatry.

[124] Alkaabba A., Hussein G., Albader M., Alosaimi B.M., Khamsah M.A., Alzahrani B., Asiri N.A., Alotaibi A.F., Al-walah M.A. (2025). The Digital Dilemma: Patterns of Screen Time, Sleep Quality, and Mental Health Among Saudi University Students. Cureus.

[125] Fabio R.A., Suriano R. (2024). The Role of Smartphone Use in Sensory Processing: Differences Between Adolescents with ADHD and Typical Development. Int. J. Environ. Res. Public Health.

[126] Fabio R.A., Suriano R. (2021). The Influence of Media Exposure on Anxiety and Working Memory during Lockdown Period in Italy. Int. J. Environ. Res. Public Health.

[127] Hattingh M., Dhir A., Ractham P., Ferraris A., Yahiaoui D. (2022). Factors Mediating Social Media-Induced Fear of Missing Out (FoMO) and Social Media Fatigue: A Comparative Study Among Instagram and Snapchat Users. Technol. Forecast. Soc. Change.

[128] Farr S.L., Dietz P.M., O’Hara M.W., Burley K., Ko J.Y. (2014). Postpartum Anxiety and Comorbid Depression in a Population-Based Sample of Women. J. Women’s Health.

